# A66 PROTON PUMP INHIBITORS AND THE RISK OF INFLAMMATORY BOWEL DISEASE: A REAL WORLD POPULATION-BASED COHORT STUDY

**DOI:** 10.1093/jcag/gwac036.066

**Published:** 2023-03-07

**Authors:** R P Yanofsky, D Abrahami, R Pradhan, H Yin, E G McDonald, A Bitton, L Azoulay

**Affiliations:** 1 Department of Gastroenterology & Hepatology, University of Toronto, Toronto, Canada; 2 Department of Medicine, Division of Pharmacoepidemiology and Pharmacoeconomics, Harvard University, Boston, United States; 3 Department of Epidemiology, Biostatistics, and Occupational Health; 4 Department of General Internal Medicine; 5 Gastroenterology & Hepatology, McGill University, Montreal, Canada

## Abstract

**Background:**

Proton pump inhibitors (PPIs) and histamine-2 receptor antagonists (H2RAs) are acid suppressant drugs indicated in highly prevalent conditions such as gastroesophageal reflux disease and peptic ulcer disease. A recent observational study reported an increased risk of inflammatory bowel disease (IBD) with the use of PPIs. However, this association may have been driven by certain methodological shortcomings, such as failure to properly account for protopathic bias, a conclusion-altering bias.

**Purpose:**

To determine whether the use of PPIs compared with the use of H2RAs is associated with an increased risk of IBD.

**Method:**

Using the longitudinal primary care records from the United Kingdom Clinical Practice Research Datalink database, we identified 1,498,416 initiators of PPIs and 322,474 initiators of H2RAs from January 1, 1990, through December 31, 2018, with follow-up until December 31, 2019. Standardized morbidity ratio weighted Cox proportional hazards models were used to estimate marginal hazard ratios (HRs) and 95% confidence intervals (CIs). Patients were analyzed according to the timing of the IBD events after treatment initiation (early versus late). In the early-event analysis, IBD events were assessed within the first two years of treatment initiation, an analysis subject to potential protopathic bias. In the late-event analysis, all exposures were lagged by two years to account for latency and minimize protopathic bias.

**Result(s):**

In the early-event analysis, the use of PPIs was associated with an increased risk of IBD within the first two years of treatment initiation, compared with H2RAs (HR: 1.39, 95% CI: 1.14-1.69). In contrast, the use of PPIs was not associated with an increased risk of IBD in the late-event analysis (HR: 1.05, 95% CI: 0.90 to 1.22). The results remained consistent in several sensitivity analyses.

**Image:**

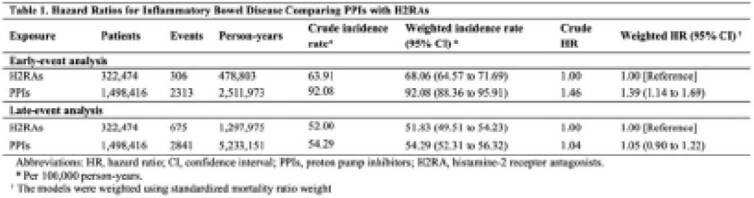

**Conclusion(s):**

Compared with H2RAs, PPIs were not associated with an increased risk of IBD, after accounting for protopathic bias.

**Please acknowledge all funding agencies by checking the applicable boxes below:**

CIHR

**Disclosure of Interest:**

R. Yanofsky: None Declared, D. Abrahami Employee of: pfizer, R. Pradhan: None Declared, H. Yin: None Declared, E. McDonald: None Declared, A. Bitton Consultant of: Member of Advisory Boards for Abbie, Pfizer, Takeda, Jansen, and Merck, Speakers bureau of: Has received speaker fees for Abbie, Jansen, Takeda, and Pfizer, L. Azoulay Consultant of: Has received consults fees from Jansen, Pfizer, and Roche, Speakers bureau of: Has received speaker fees from Jansen, Pfizer, and Roche

